# Development of a standardized histopathology scoring system for intervertebral disc degeneration and regeneration in rabbit models‐An initiative of the ORSspine section

**DOI:** 10.1002/jsp2.1147

**Published:** 2021-06-03

**Authors:** Sarah E. Gullbrand, Beth G. Ashinsky, Alon Lai, Jennifer Gansau, James Crowley, Carla Cunha, Julie B. Engiles, Marion Fusellier, Carol Muehleman, Matthew Pelletier, Steven Presciutti, Jordy Schol, Yoshiki Takeoka, Takashi Yurube, Yejia Zhang, Koichi Masuda, James C. Iatridis

**Affiliations:** ^1^ University of Pennsylvania Philadelphia Pennsylvania USA; ^2^ Corporal Michael J. Crescenz VA Medical Center Philadelphia Pennsylvania USA; ^3^ Leni and Peter W. May Department of Orthopaedics Ichan School of Medicine New York New York USA; ^4^ Surgical and Orthopaedic Research Laboratories, Prince of Wales Clinical School UNSW Sydney Australia; ^5^ i3S ‐ Instituto de Investigação e Inovação em Saúde, INEB ‐ Instituto de Engenharia Biomédica Porto Portugal; ^6^ Department of Clinical Studies, New Bolton Center School of Veterinary Medicine, University of Pennsylvania, Philadelphia Pennsylvania USA; ^7^ Inserm, UMR 1229, RMeS, Université de Nantes, ONIRIS Nantes France; ^8^ Rush University Medical Center Chicago Illinois USA; ^9^ Emory University Atlanta Georgia USA; ^10^ Tokai University School of Medicine Isehara Japan; ^11^ Brigham and Women's Hospital Boston Massachusetts USA; ^12^ Kobe University Graduate School of Medicine Kobe Japan; ^13^ University of California San Diego California USA

**Keywords:** histology, histology grading scale, histopathology, intervertebral disc degeneration, intervertebral disc regeneration, rabbit

## Abstract

**Background:**

The rabbit lumbar spine is a commonly utilized model for studying intervertebral disc degeneration and for the pre‐clinical evaluation of regenerative therapies. Histopathology is the foundation for which alterations to disc morphology and cellularity with degeneration, or following repair or treatment are assessed. Despite this, no standardized histology grading scale has yet been established for the spine field for any of the frequently utilized animal models.

**Aims:**

The purpose of this study was to establish a new standardized scoring system to assess disc degeneration and regeneration in the rabbit model.

**Materials and Methods:**

The scoring system was formulated following a review of the literature and a survey of spine researchers. Validation of the scoring system was carried out using images provided by 4 independent laboratories, which were graded by 12 independent graders of varying experience levels. Reliability testing was performed via the computation of intra‐class correlation coefficients (ICC) for each category and the total score. The scoring system was then further refined based on the results of the ICC analysis and discussions amongst the authors.

**Results:**

The final general scoring system involves scoring 7 features (nucleus pulposus shape, area, cellularity and matrix condensation, annulus fibrosus/nucleus pulposus border appearance, annulus fibrosus morphology, and endplate sclerosis/thickening) on a 0 (healthy) to 2 (severe degeneration) scale. ICCs demonstrated overall moderate to good agreement across graders. An addendum to the main scoring system is also included for use in studies evaluating regenerative therapeutics, which involves scoring cell cloning and morphology within the nucleus pulposus and inner annulus fibrosus.

**Discussion:**

Overall, this new scoring system provides an avenue to improve standardization, allow a more accurate comparison between labs and more robust evaluation of pathophysiology and regenerative treatments across the field.

**Conclusion:**

This study developed a histopathology scoring system for degeneration and regeneration in the rabbit model based on reported practice in the literature, a survey of spine researchers, and validation testing.

## Graphical abstract

The rabbit lumbar spine is a commonly utilized model for studying intervertebral disc degeneration and for the pre‐clinical evaluation of regenerative therapies, and histopathology is the foundation for which alterations to disc morphology and cellularity with degeneration, or following repair or treatment are assessed. Despite this, no standardized histology grading scale has yet been established for the spine field for any of the frequently utilized animal models. Therefore, the purpose of this study was to establish a new standardized scoring system to assess disc degeneration and regeneration in the rabbit model.

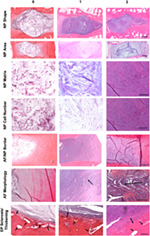

## 1 INTRODUCTION

The intervertebral discs of the spine are the composite, avascular structures which reside between bony vertebral bodies and are responsible for bearing often high magnitude loads derived from complex spinal motion during activities of daily living.^1^, ^2^ The intervertebral disc is composed of the highly hydrated, proteoglycan‐rich nucleus pulposus (NP), surrounded by the annulus fibrosus (AF), which is composed of lamellae of primarily type I collagen fibers with alternating ±30° orientation to the transverse plane.^3^ Bounding the disc both superiorly and inferiorly are the bony and cartilaginous endplates (EPs), which play a key role in constraining the NP and regulating nutrient transport to and from the avascular disc.^4^, ^5^

Intervertebral disc degeneration is associated with a variety of structural, compositional, and mechanical perturbations to all of the disc substructures.^6^ Degeneration of the intervertebral discs is considered to be a major contributor to back pain, which has become a primary cause of disability globally.^7^, ^8^ Studying the etiology and progression of disc degeneration in humans can be difficult, costly, and time consuming, and as such, animal models are commonly used to study the progression of this disease or to evaluate the efficacy of new therapeutic interventions to affect disc regeneration.^9^, ^10^ The rabbit lumbar spine is a common preclinical model to study disc degeneration and to evaluate therapeutic interventions.^11^, ^12^ The rabbit model has the advantage of cost‐effectiveness compared to larger animal models (ie, canine, sheep, goats), while possessing comparable anatomy to humans because of the presence of facet joints, paravertebral muscles and ligaments compared to the commonly utilized rodent tail models. Compared to preclinical rodent tail models, the larger size of rabbits means surgical intervention can be more precise and device implantation is possible. Normalized mechanical properties of the rabbit motion segment measured in axial compression and torsion are similar to human discs, as are the glycosaminoglycan content of the NP and AF.^13^ Geometric properties of the rabbit lumbar disc, including disc height, dorso‐ventral width, and NP area deviate by 26% from human geometry, after normalization.^14^ Limitations of the rabbit model include the persistence of notochordal cells into adulthood, in contrast to humans where the notochordal cell population is largely lost in adolescence.^15^ Additionally, unlike mouse and rat models, well established methods to assess pain in rabbit models of disc degeneration do not yet exist.

Histology is a common experimental endpoint for studies of disc degeneration and regeneration, yet standardized histopathology grading systems for animal models and humans do not yet exist for the spine field as they do, for example, in the cartilage field with the Osteoarthritis Research Society International (OARSI) score.^16^ The lack of widespread adoption of a histologic scoring system in the field renders comparison of results across studies from multiple groups difficult. The purpose of this work was to establish and validate a standardized histological scoring system for grading intervertebral disc degeneration and regeneration in the rabbit model. To do so, we performed three studies: (a) a literature review of published papers utilizing the rabbit model, (b) a survey of spine researchers, and (c) validation of the new scoring system.

## 2 METHODS

### 2.1 Literature review

A Pubmed search through 31 December 2019 was conducted using the search term “intervertebral disc degeneration rabbit model.” Papers not published in English were excluded, as were papers with no in vivo component, such as those utilizing computational methods, or in vitro or ex vivo cell or organ culture models. The full text portable document formats of the included studies were obtained and reviewed for the type of histology performed, the stains utilized, if histology grading was performed and what grading scheme was used, and what additional outcome measures were reported.

### 2.2 Survey study

Based on the results of the literature review, a survey was generated in Google forms to query spine researchers regarding their current practices regarding histological processing of rabbit discs and their opinion on which categories would be important to include in a consensus scoring system. A copy of the survey is included in the supplemental information. (Supplemental Data 1) A survey link was sent via e‐mail to current active members of the Orthopedic Research Society (ORS) Spine Section, and the corresponding authors of the 50 most recent papers published using the rabbit model (as identified from the literature review).

### 2.3 Scoring system validation and refinement

Based on the literature review and the survey results, a scoring system for disc degeneration and regeneration was drafted (Table 1). The scoring system for degeneration consisted of six categories (NP morphology, NP matrix, AF/NP border, AF, EP, NP cellularity), scoring seven features (NP shape, NP area, NP matrix condensation, NP cell number, AF/NP border appearance, AF morphology, EP sclerosis/thickening) on a three point scale (0‐2; Table 1). NP cell number was replaced in the regeneration scoring system with cell cloning and cell morphology within the NP and inner AF. The degeneration scoring system was designed for use in studies investigating only a degenerative response, such that a higher score indicates a more degenerative disc. The regeneration scoring system was designed for use in studies investigating a regenerative therapeutic in comparison to a sham treatment (ie, saline injection), such that a higher score would indicate a more degenerative disc with no repair, while a lower score would be indicative of repair processes or a healthier disc.

**TABLE 1:** The scoring system drafted after review of the literature and collation of the survey responses, which was utilized for the validation studies

	Category	Features	Score	Description	Reference
Degeneration AND regeneration studies	NP morphology	NP shape	0	Oval shape	Han, 2008^21^ Mao, 2011^22^
			1	Oval/round with mild distortion	
			2	Irregular shape	
		NP area	0	NP constitutes more than 50% of disc area	
			1	NP constitutes between 25% and 50% of disc area	
			2	NP constitutes less than 25% of disc area	
	NP matrix	Matrix condensation	0	Normal gelatinous appearance	Masuda, 2005
			1	Slight condensation of the extracellular matrix	
			2	Moderate/severe condensation of the extracellular matrix	
	AF/NP border	Border appearance	0	Normal, clear distinction between NP and AF	Han, 2008^21^ Masuda, 2005^11^
			1	Minimally interrupted, loss of distinction between NP and AF	
			2	No distinction between NP and AF	
	AF	AF morphology	0	Normal, pattern of fibrocartilage lamellae (u‐shaped posteriorly and slightly convex anteriorly), without ruptured fibers and without serpentine appearance anywhere in AF	Masuda, 2005^11^
			1	Ruptured or serpentine fibers in less than 30% of AF, mild/moderate infolding	
			2	Ruptured or serpentine fibers in more than 30% of AF, severe infolding	
	EP	EP sclerosis/thickening	0	No bony sclerosis, thin cartilage endplate	Ashinsky, 2020^20^
			1	Mild bony sclerosis and thickening of the cartilage endplate	
			2	Severe sclerosis and thickening of the cartilage endplate	
Degeneration only studies	NP cellularity	Cell number	0	Normal cell number	Masuda, 2005^11^
			1	Mild/moderate reduction in number of cells	
			2	Severe reduction in number of cells	
Regeneration studies	NP and inner AF repair	Cell cloning	0	Extensive cell cloning	Chujo, 2006^18^
			1	Mild/moderate cell cloning	
			2	No cell clones	
		Cell morphology	0	Many large, rounded cells with intense pericellular matrix staining	
			1	Few large, rounded cells with intense pericellular matrix staining	
			2	No large, rounded cells with intense pericellular matrix staining	

Abbreviations: AF, annulus fibrosus; EP, end plate; NP, nucleus pulposus.

Rabbit histopathology images were collected from four groups in the field who have used the rabbit model to study disc degeneration (n = 22 slides, from injury models such as puncture or nucleotomy) or to evaluate repair or regeneration (n = 19 slides, from growth factor treatment studies). Slides were sagittal sections stained with Hematoxylin and Eosin (H&E), Alcian Blue and Picrosirius Red, or Safranin‐O and Fast Green. Images were collected, blinded, and distributed to 15 individual graders for scoring. Graders were asked to self‐classify as “expert” (having at least 2 years of experience with rabbit histopathology grading) or “novice”. Scoring was completed by five expert and seven novice graders. Graders scored the images twice, with 1 week in between scoring sessions to assess intra‐rater reliability. Intra‐ and inter‐rater reliability for each scoring category, as well as total score, were assessed by calculating intraclass correlation coefficients (ICC) in R (https://www.r-project.org/). ICC values above 0.9 were considered to indicate excellent agreement, whereas values between 0.75 and 0.9 were considered to indicate good agreement, values between 0.5 and 0.75 moderate agreement, and values below 0.5 poor agreement.^17^ Based on the ICC results from the draft scoring system, the language and example images in the scoring system were further refined.

### 2.4 Ethics statement

For the survey study, the distribution and collection of the survey was deemed exempt research by the Corporal Michael J. Crescenz Veterans Affairs (VA) Medical Center Institutional Review Board (Protocol #01862). The study conforms to the US Federal Policy for the Protection of Human Subjects. No live animals were used for this study on rabbits. This study complies with US National Research Council's Guide for the Care and Use of Laboratory Animals, the US Public Health Service's Policy on Humane Care and Use of Laboratory Animals, and Guide for the Care and Use of Laboratory Animals.

## 3 RESULTS

### 3.1 Literature review

**FIGURE 1:**
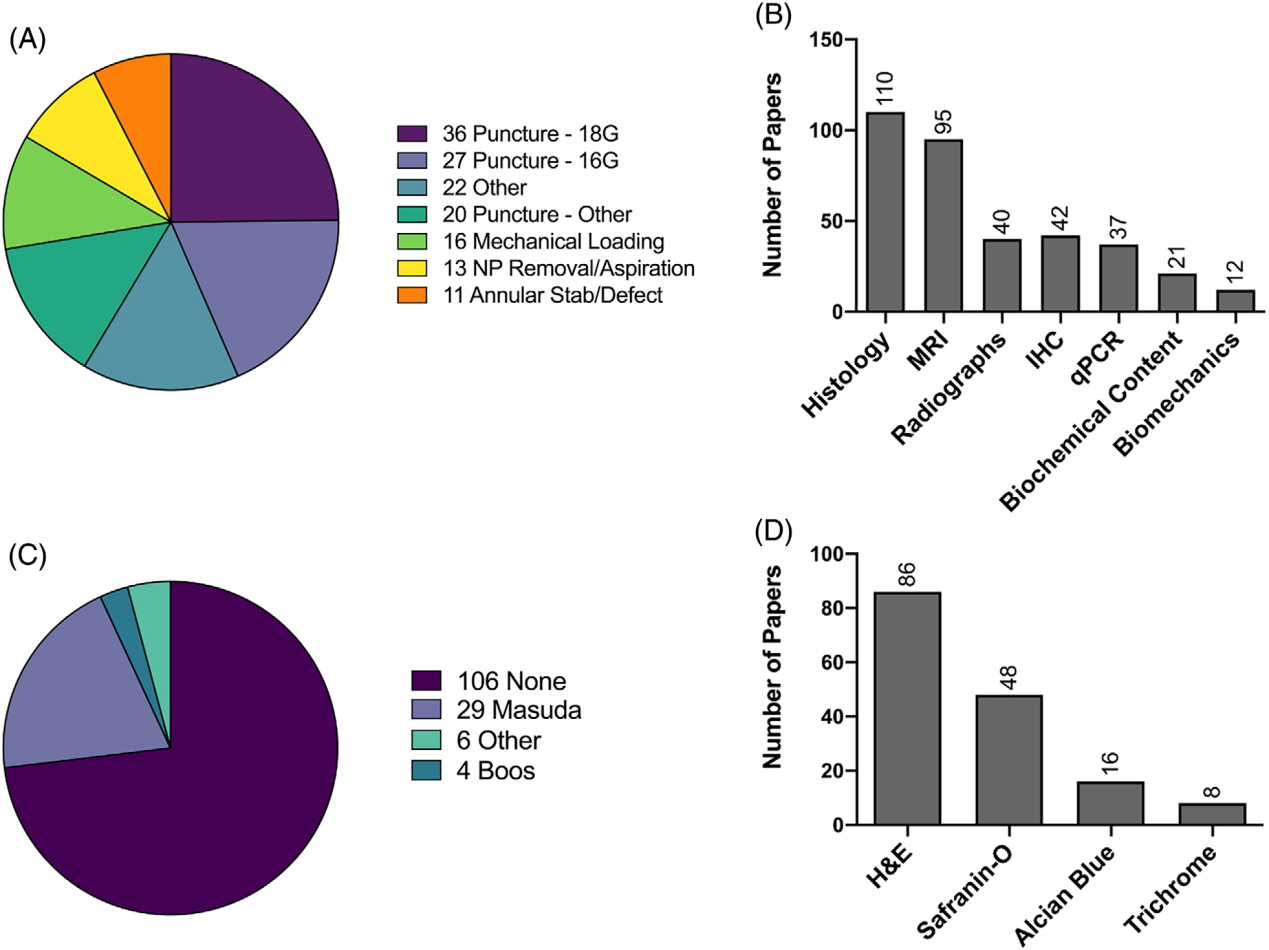
Results of the literature review summarizing the number of papers (as indicated next to the color legend) utilizing different A, mechanisms of inducing disc degeneration; B, metrics for assessing disc degeneration or regeneration; C, existing histology scoring systems; and D, histologic stains

The initial PubMed search yielded 236 manuscripts. Ninety‐one papers fell within the exclusion criteria, and the remaining 145 papers were further reviewed. This literature search identified several methods of inducing disc degeneration in the rabbit model (Figure 1A); the most common of which was via needle puncture, which accounted for 60% of all papers reviewed. Within the puncture models, the most common needle gauge utilized to induce degeneration was an 18G needle (26% of papers). Additional common methods of inducing degeneration included annular stab or defect creation, NP removal or aspiration, or mechanical loading. Less frequently used models, included in the “other” category, were EP disruption or injury, nicotine administration, aging, gene knockout, chemonucleolysis, or injection of a catabolic agent.

For model outcomes, approximately 76% of papers included histology as an outcome measure (Figure 1B). The most common stains utilized for histology in the rabbit model included H&E, and Safranin‐O and Fast Green staining. Clinically relevant imaging methods, including magnetic resonance imaging (MRI) and radiographs were the next commonly used metrics for assessing degeneration or regeneration. Only 8% of papers included some form of biomechanical testing as an outcome measure. Despite the prevalence of histological analyses in rabbit degeneration models, only 27% of papers performed semi‐quantitative histological grading of their samples (Figure 1C). Of the papers which performed histological grading, the grading scheme proposed by Masuda et al was most commonly used.^11^ This grading scheme utilizes a 12 point scale, consisting of categories of “annulus fibrosus,” “border between the annulus fibrosus and nucleus pulposus,” “nucleus pulposus cellularity,” and “nucleus pulposus matrix”. In the Masuda scoring system, each category is scored from 1 to 3 points, such that a minimal score of 4 represents no degenerative changes and a maximum score of 12 represents severe degeneration.

### 3.2 Survey study

**FIGURE 2:**
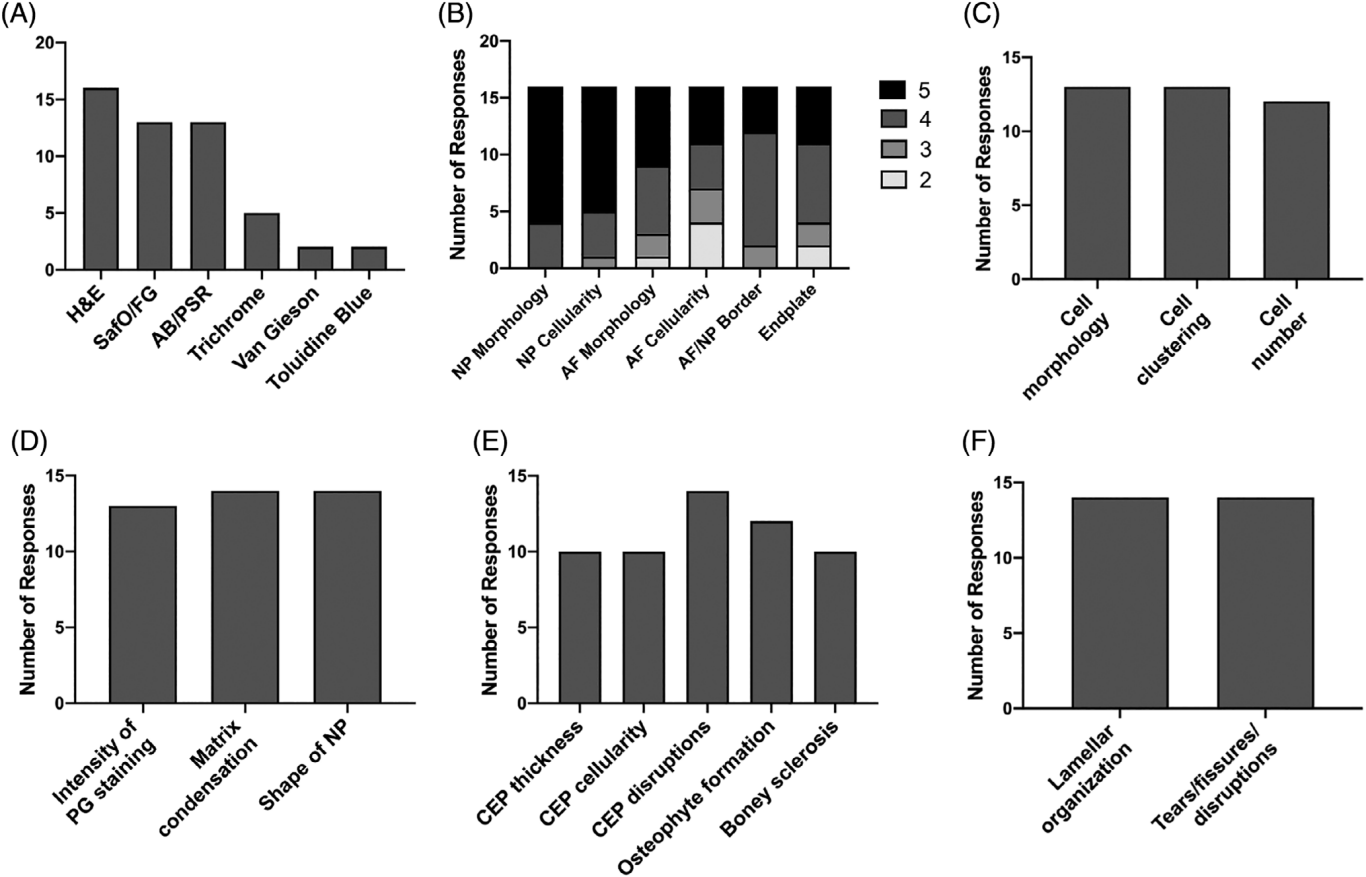
Results of the survey of spine researchers indicating opinions on which A, histology stain should be used for rabbit histopathology scoring and B, importance (1 = low importance, 5 = high importance) of including different categories in the new scoring system. Survey respondents were asked to select all features they considered important to consider when scoring, within the broad categories of C, AF/NP cellularity; D, NP morphology,; E, Endplate; and F, AF morphology

Sixteen responses were received for the rabbit histopathology survey. Survey respondents indicated that paraffin processing (87.5% of respondents) should be utilized for rabbit spine histology, with discs sectioned in the sagittal plane (75% of respondents) at <10 μm thickness (100% of respondents). H&E staining (100% of respondents) or Safranin‐O and Fast Green (81% of respondents) or Alcian Blue and Picrosirius Red (81% of respondents) were selected most frequently as the stains which should be used, similar to the literature review findings (Figure 2A). Next, respondents were asked to rank categories for histology scoring on a 1 (least important) to 5 (most important) scale. Over 50% of respondents ranked the categories of NP morphology, NP cellularity, AF morphology, AF‐NP border and EP as a 4 or 5. AF cellularity was ranked as the category with lowest importance, with more than 43.8% of respondents rating this category a 2 or 3 (Figure 2B). When asked to designate important features which should be included within each scoring category, most of the features provided as options were selected by respondents (Figure 2C‐F).

### 3.3 Recommendations for processing and use of the scoring system

**FIGURE 3:**
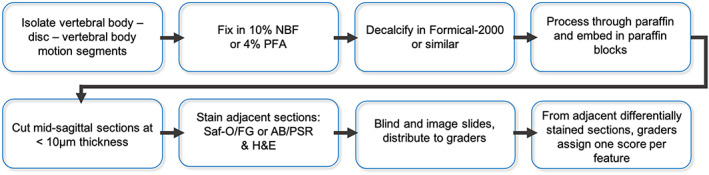
Flow chart for recommended processing methods and implementation of the scoring system. AB/PSR, Alcian blue/picrosirius red; H&E, hematoxylin & eosin; NBF, neutral buffered formalin; PFA, paraformaldehyde; Saf‐O/FG, Safranin‐O and Fast Green

**FIGURE 4:**
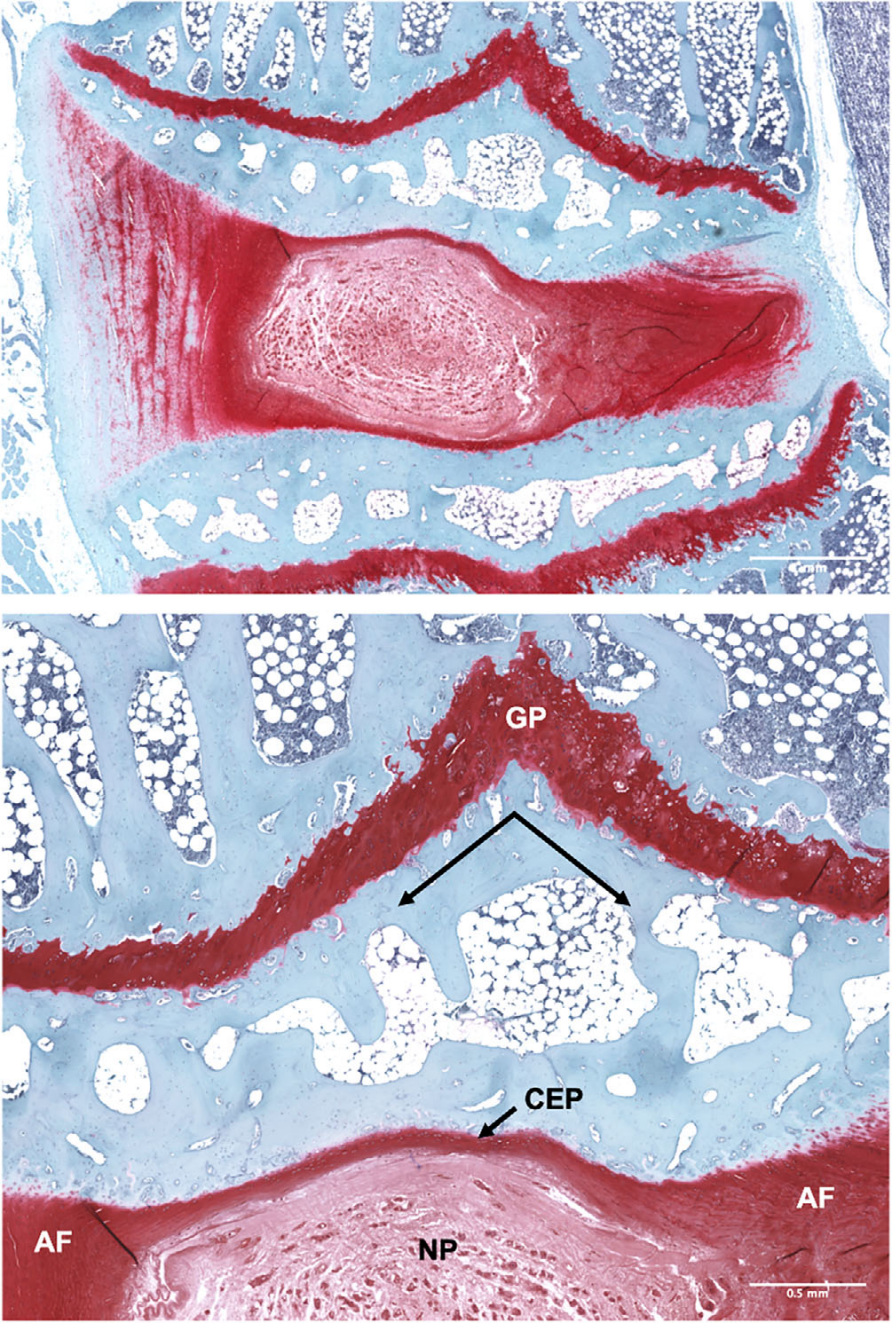
Sagittal section of a rabbit lumbar intervertebral disc stained with Safranin‐O and Fast Green, demonstrating the central, gelatinous nucleus pulposus (NP) surrounded by the collagenous, laminar annulus fibrosus (AF) and cartilaginous endplates (CEP) with the characteristic vee shape (arrows) of the growth plate (GP) in the mid‐sagittal plane. Top scale = 1 mm, bottom scale = 500 μm

To increase the consistency between studies across groups, we propose recommendations for histology processing for rabbit spinal motion segments, based on our review of common practices in the literature and current spine researchers (Figure 3). We recommend that paraffin processing in the sagittal plane be utilized to obtain sections for use with the scoring system, as it allows for the acquisition of many, high quality, thin sections most ideal for semi‐quantitative histologic scoring. This generally involves fixation of the bone‐intervertebral disc‐bone motion segment (4% paraformaldehyde or 10% neutral buffered formalin), followed by decalcification and processing through paraffin. Mid‐sagittal paraffin sections should be cut using a microtome at a thickness < 10 μm. The midsagittal plane in the rabbit motion segment is identifiable based on the characteristic vee shape of the growth plate above the NP (Figure 4). We recommend that slides utilized for histologic scoring be stained with H&E to visualize cell morphology. Additional adjacent sections should be stained with either Alcian Blue and Picrosirius Red or Safranin‐O and Fast Green to visualize proteoglycan and collagen distribution. Ideally slides should be scanned on a slide scanner to generate tile scans at ×20 to ×40x magnification, such that graders can visualize macroscopic morphology, then zoom in to appreciate cell morphology on the same slide. If a slide scanner is unavailable, macroscopic (2‐4×) images, in addition to higher magnification (20x) images of the different regions of the disc (CEP, NP, AF) should be provided to graders. The pair of differentially stained adjacent sections should be utilized simultaneously to assign a single score (0‐2) for each morphologic feature of the scoring system for each sample. Alternately, Safranin‐O and Fast Green stained sections can be counterstained with Hematoxylin to visualize cell distribution and morphology, as well as proteoglycan content within the same sample. Scores may be reported by category if desired, in addition to reporting total score by summing the scores for each feature. We recommend slides be graded by raters who are blinded to the experimental groups.

### 3.4 Scoring system validation and refinement

For the initial proposed scoring system (Table 1), the ICC values and 95% confidence intervals (CIs) are listed in Table 2 for each scoring category, separated by expert and novice graders. Generally, there was better agreement between expert graders compared to novice graders. Inter‐observer ICC values were also generally higher for degeneration scoring compared to regeneration, despite significant overlap in the categories in the scoring systems for degeneration and regeneration. Moderate (0.5 < ICC < 0.75) to good (0.75 < ICC < 0.9) agreement between graders was found for most scoring categories. Categories with consistently poor agreement (ICC < 0.5) by both expert and novice graders included EP sclerosis/thickening for both degeneration and regeneration scoring systems, AF morphology for the regeneration scoring system, and NP/inner AF cell cloning and cell morphology for the regeneration scoring system. Intra‐observer reliability indicated that the reproducibility for using this scoring system was excellent among all graders, with ICCs ranging from 0.81 (95% CI: 0.59‐0.92) to 0.99 (0.97‐1.0).

**TABLE 2:** Intraclass correlation coefficients and 95% confidence intervals (listed in parentheses) for the degeneration and regeneration scoring systems across seven novice graders and five expert graders

	Degeneration		Regeneration	
	Novice	Expert	Novice	Expert
Total score	0.78	0.82	0.59	0.76
	(0.64‐0.88)	(0.70‐0.91)	(0.40‐0.77)	(0.59‐0.88)
NP shape	0.75	0.73	0.40	0.70
	(0.61‐0.87)	(0.58‐0.86)	(0.22‐0.64)	(0.51‐0.844)
NP area	0.68	0.64	0.31	0.53
	(0.52‐0.83)	(0.47‐0.80)	(0.14‐0.56)	(0.33‐0.74)
NP matrix	0.73	0.84	0.53	0.76
	(0.58‐0.86)	(0.73‐0.92)	(0.34‐0.74)	(0.60‐0.88)
AF/NP border	0.66	0.74	0.48	0.71
	(0.49‐0.81)	(0.60‐0.87)	(0.29‐0.70)	(0.54‐0.85)
AF morphology	0.54	0.47	0.10	0.23
	(0.36‐0.73)	(0.28‐0.68)	(‐0.04‐0.34)	(0.05‐0.49)
EP sclerosis/thickening	0.10	0.38	0.03	0.24
	(‐0.02‐0.30)	(0.20‐0.61)	(‐0.07‐0.22)	(0.06‐0.50)
NP cell number	0.41	0.52	—	—
	(0.23‐0.62)	(0.33‐0.72)		
NP/inner AF cell cloning	—	—	0.37	0.19
			(0.19‐0.61)	(0‐0.47)
NP/inner AF cell morphology	—	—	0.55	0.14
			(0.36‐0.75)	(‐0.05‐0.41)

Abbreviations: AF, annulus fibrosus; EP, end plate; NP, nucleus pulposus.

**FIGURE 5:**
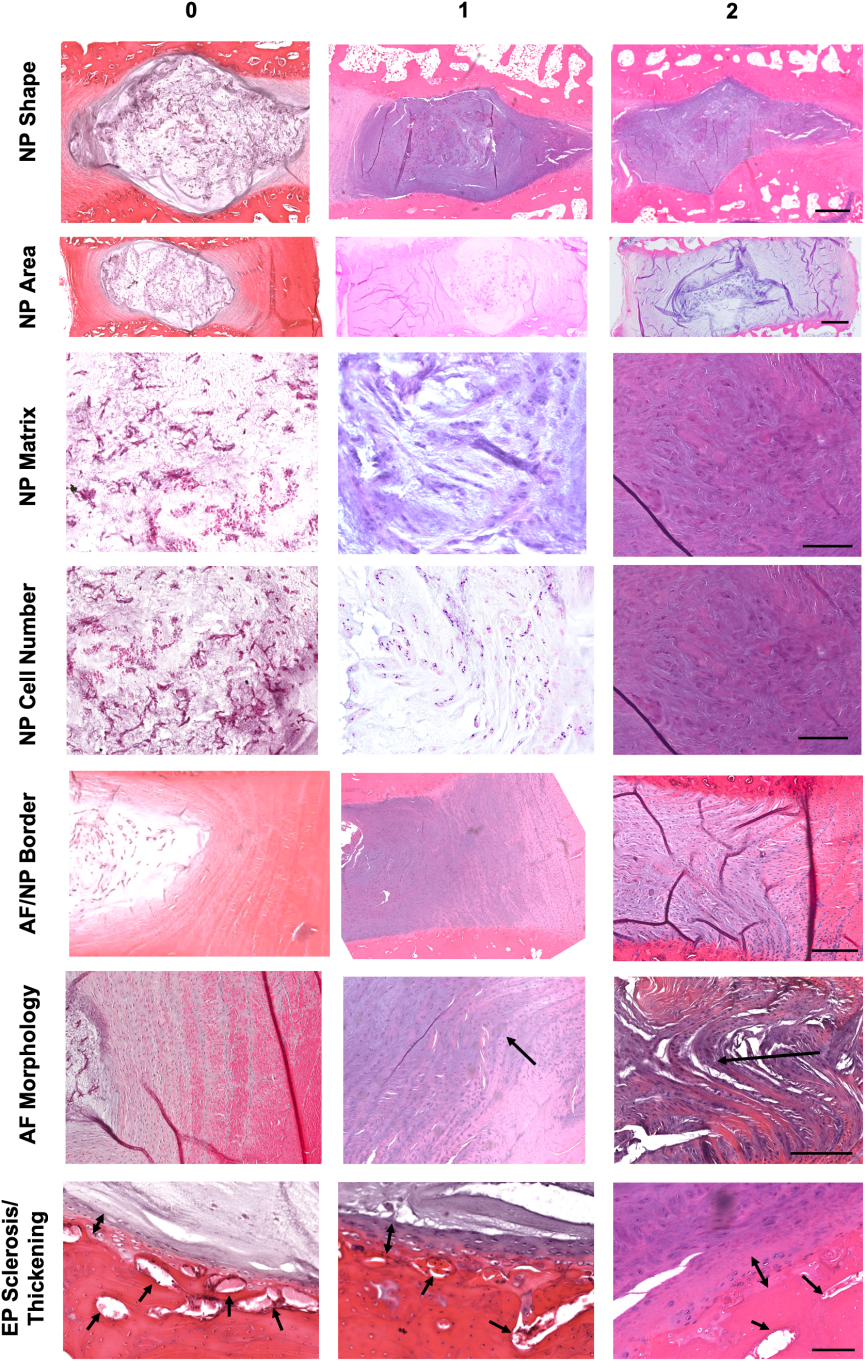
Example images depicting the morphologic changes within each category of the final main scoring system. Arrows in the annulus fibrosus (AF) morphology panel indicate infolding of AF lamellae into the nucleus pulposus (NP). Double‐headed arrows in the end plate (EP) sclerosis/thickening panel indicate the increasing thickness of the cartilage endplate, and single‐headed arrows indicate a reduction in the number of vascular channels. Scales = 500 μm for NP shape, NP area, AF/NP border, AF morphology. Scale = 100 μm for EP sclerosis/thickening. Scales = 200 μm for NP matrix and cell number

**FIGURE 6:**
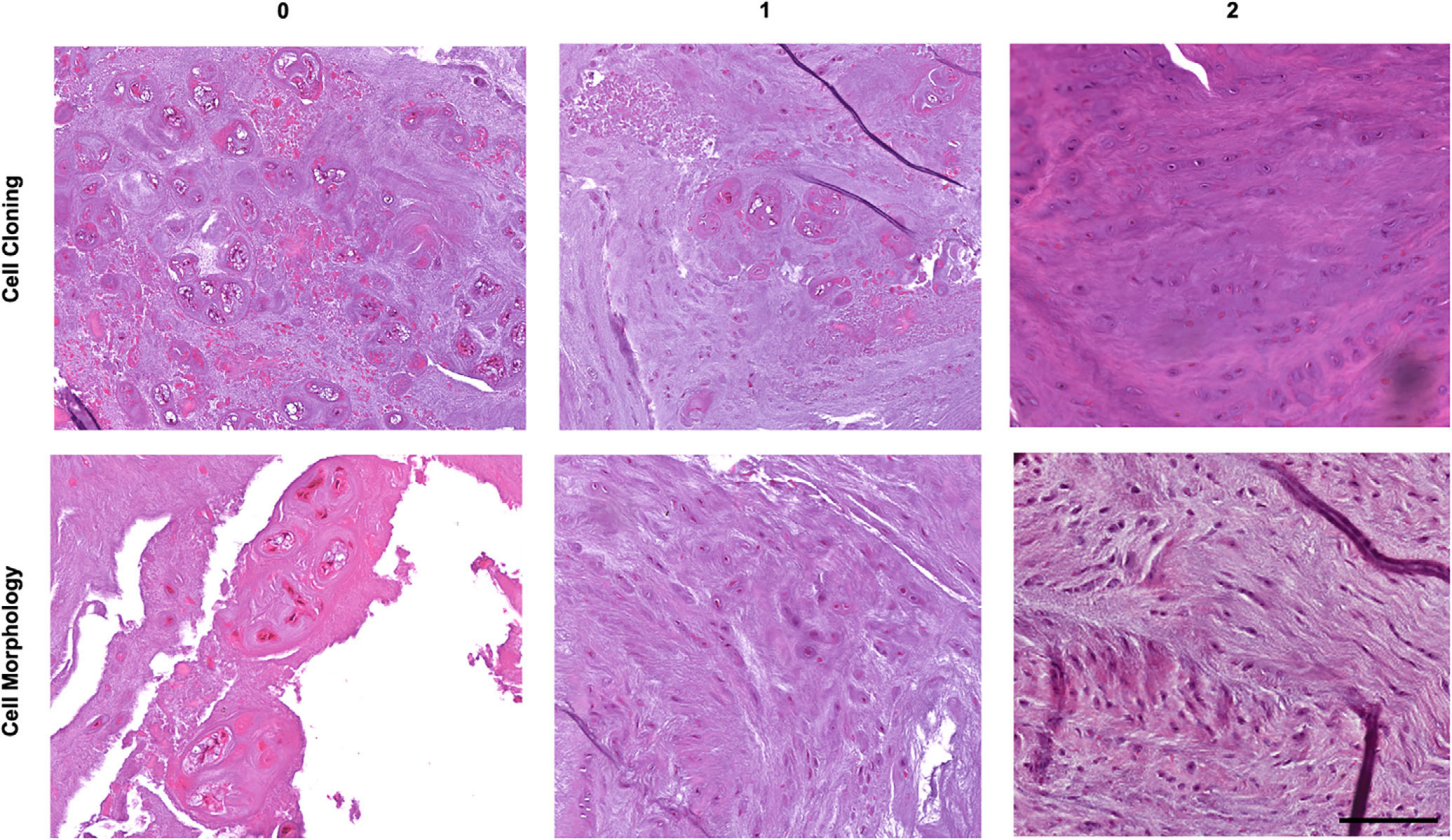
Example images depicting morphologic changes within each category for the addendum repair scoring system. Scale = 200 μm

Based on the ICC results, and following discussion among the authors, the language of the scoring systems and the example images provided were refined to further clarify the features to be scored in the categories with poor ICC and maximize the usability of the scoring system. The final scoring system is summarized in Table 3 and example images most representative for each feature are provided in Figure 5. This main scoring system, yielding total scores of 0 (healthy) to 14 (severely degenerative), may be utilized for any study in the rabbit model, including induced degeneration and regeneration studies. The cellular morphology observed during regeneration is typically characterized by extensive cell cloning (clusters of 4 or more cells), where cells have a rounded morphology with intense pericellular matrix staining.^18^ This regenerative response is characterized by inherently different cellular morphology than observed in a normal, healthy rabbit disc, and thus cannot be captured with the same scoring system as for changes with degeneration compared to healthy controls. Therefore, we also propose an addendum to the main scoring system to be utilized only in studies involving comparison of a regenerative treatment to a sham treatment (Table 4, Figure 6). This repair score, yielding total scores of 0 (robust repair) to 4 (no repair), can be reported independently from the main score, or summed with the main score, yielding total scores from 0 to 18.

**TABLE 3:** The final main scoring system after refinement based upon the grader reliability analysis and discussion amongst the authors, to be used for studies of both degeneration and regeneration

Category	Features	Score	Description	Reference
NP monophology	NP shape	0	Oval shape	Han, 2008^21^ Mao, 2011^22^
		1	Oval/round with mild distortion	
		2	Irregular shape	
	NP area	0	NP constitutes 40‐50% of disc area	
		1	Mild to moderate reduction in NP area	
		2	Severe reduction in NP area	
NP matrix	Matrix condensation	0	Normal gelatinous appearance	Masuda, 2005^11^
		1	Slight condensation of the extracellular matrix	
		2	Moderate/severe condensation of the extracellular matrix	
NP cellularity	Cell number	0	High cell density	Masuda, 2005^11^
		1	Medium cell density	
		2	Low cell density	
AF/NP border	Border appearance	0	Normal, clear distinction between NP and AF	Han, 2008^21^ Masuda, 2005^11^
		1	Minimally interrupted, loss of distinction between NP and AF	
		2	No distinction between NP and AF	
AF	AF morphology	0	Normal, pattern of fibrocartilage lamellae (u‐shaped posteriorly and slightly convex anteriorly), without ruptured fibers and without serpentine appearance anywhere in AF	Masuda, 2005^11^
		1	Ruptured or serpentine fibers in less than 30% of AF, mild/moderate infolding	
		2	Ruptured or serpentine fibers in more than 30% of AF, severe infolding	
EP	EP sclerosis/thickening	0	Thin cartilage endplate with many adjacent vascular/marrow channels.	Ashinsky, 2020^20^
		1	Mild/moderate thickening of cartilage endplate and reduction in number of adjacent vascular/marrow channels	
		2	Severe thickening of the cartilage endplate and reduction in number of adjacent vascular/marrow channels	

Abbreviations: AF, annulus fibrosus; EP, end plate; NP, nucleus pulposus.

**TABLE 4:** The repair scoring system to be used as an addendum to the main scoring system for studies evaluating regenerative therapeutics

Category	Features	Score	Description	Reference
NP and inner AF repair	Cell cloning	0	Extensive cell cloning	Chujo, 2006^18^
		1	Mild/moderate cell cloning	
		2	No cell clones	
	Cell morphology	0	Many large, rounded cells with intense pericellular matrix staining	
		1	Few large, rounded cells with intense pericellular matrix staining	
		2	No large, rounded cells with intense pericellular matrix staining	

Abbreviations: AF, annulus fibrosus; NP, nucleus pulposus.

## 4 DISCUSSION

In this study, we have proposed a new standardized histopathology scoring system to assess disc degeneration and regeneration in the rabbit lumbar spine, based on previous literature and current practices by research groups in the field. Seventy‐three percent of previously published papers using rabbit models for degeneration or regeneration did not perform histologic scoring in their studies, highlighting the significant need for a simple, standardized histopathology scoring system for rabbit models. As a majority of studies in the rabbit model that perform histology scoring have utilized the grading scheme proposed in 2005 by Masuda et al,^11^, ^19^ this scoring system was utilized as the basis of the proposed scoring system. The Masuda scoring system for disc degeneration included categories for characterizing changes to the AF and NP, but did not include scoring of the EP to assess cartilage and bony EP remodeling, or scoring for cellular changes occurring with regeneration which provides information on the biological action of regenerative strategies. Scoring of these features was therefore included in the proposed scoring system, modified from scoring systems utilized by Chujo et al^18^ and Ashinsky et al.^20^ Because the use of decalcified, paraffin processed sections is recommended for scoring, the endplate features included in the scoring system are focused solely on morphologic changes to this region. Researchers interested in studying alterations in calcification of this region would need to employ alternative methods, such as microcomputed tomography or cryo‐ or plastic sectioning to quantify or visualize endplate calcification.

Additional categories for grading NP morphology changes (area and shape), previously utilized in the rat model,^21^, ^22^ were also included to further increase the resolution of the scoring system for assessing degeneration and regeneration, as NP morphology was ranked by survey respondents as the most important category to be included in a histology grading scheme (Figure 2). Additionally, in the Masuda scoring system, a disc with no degeneration would have a total score of 4, which is less intuitive than a score of 0 for a healthy disc as utilized in the proposed scoring system. The proposed scoring system also allows researchers to quantify more subtle histological changes compared to scoring systems based solely on gross morphology, such as the Thompson grade.^23^ Overall, the proposed scoring system provides the capacity to quantify changes across the whole motion segment; however, depending on each particular research question being asked in the rabbit model, adaptation of the scoring system might be necessary to exclude categories not relevant to the study design, or further expand upon existing categories.

Validation of the scoring system was performed by 12 independent graders of varying experience levels, on images collected from different laboratories. No training was given to graders beyond requesting they familiarize themselves with the language of the scoring system and the example images provided prior to scoring the images. ICC values for total degeneration or regeneration score indicated good agreement across all graders, and most individual scoring categories had moderate to good agreement between graders. However, there were certain categories with poor agreement, namely the endplate and cell number and morphology categories. This may be explained by the more subtle changes with degeneration that occur in these structures, and for novice graders, lack of familiarity with the normal anatomy of the rabbit motion segment. Overall however, the ICC values reported in this study are generally within the range of those reported for the OARSI scoring system developed in rat (ICCs ranging from 0.44 to 0.99 depending on category and experience level of grader) and rabbit (ICCs ranging from 0.82 to 0.99 depending on category and experience level of grader).^16^, ^24^

These results also point to a need for more extensive training of graders prior to utilizing any histopathology scoring system. Research groups may wish to consider developing a “training set” of images obtained from their laboratory, which can demonstrate to graders how the features to be scored present in their groups' model, and with their processing methods. Future efforts by this group will involve the development of virtual content and workshops that may be utilized for training new users of the scoring system. An additional approach which may be considered is scoring by consensus, as has been performed in OARSI scoring of human samples,^22^ whereby scoring is conducted as a group and a consensus score is agreed upon by all graders for each image.

The scoring system proposed here is designed to serve as a first step towards standardized outcomes in disc degeneration and regeneration studies in the rabbit model to allow for more accurate comparison between labs and more robust evaluation of pathophysiology and regenerative treatments. We not only anticipate future refinement of the histopathology scoring system itself, but also the establishment of standards for other outcome metrics commonly utilized to probe changes with degeneration and regeneration in animal models. Survey respondents indicated the most interest in future methods papers to improve standardization in the field on the topics of MRI (14 responses), radiographic measurement of disc height (11 responses) and biochemical assays to quantify glycosaminoglycan and collagen content (13 responses). MRI and radiographic analyses in the rabbit model have been commonly reported in the literature; however, only a minority of studies utilize biomechanical or biochemical outcomes (Figure 1). Future manuscripts providing guidance towards standardizing these metrics could help further their more widespread adoption by the field. Additionally, it would be beneficial to generate consensus on which method of inducing disc degeneration in the rabbit model yields morphologic changes most closely resembling the human condition. Scoring systems help support more common language and methods, yet are expected to continue to evolve with the addition of new perspectives, and innovations in scientific thought and methods.

In conclusion, we have developed a new standardized histopathology scoring system for intervertebral disc degeneration and regeneration in the rabbit lumbar spine model. This scoring system was developed based on prior literature as well as input from spine researchers using this model, and was validated with images across multiple laboratories by 12 independent graders. It represents one of several parameters utilized in the assessment of intervertebral disc health in an animal model. As this scoring system is further validated and refined, it will facilitate a more objective comparison of results across laboratories, degeneration models, and regenerative therapies.

## CONFLICT OF INTEREST

The authors declare no potential conflicts of interest.

## AUTHOR CONTRIBUTIONS

Conception and design of the study: Sarah E. Gullbrand, Alon Lai, Jennifer Gansau, James C. Iatridis; Literature review and analysis: Sarah E. Gullbrand, James C. Iatridis; Survey drafting and analysis of responses: Sarah E. Gullbrand, Alon Lai, Jennifer Gansau, James C. Iatridis; Provided input on preliminary scoring system development: Sarah E. Gullbrand Koichi Masuda, Carol Muehleman, James C. Iatrids; Provided histological images: Sarah E. Gullbrand, Koichi Masuda, Steven Presciutti; Critical review and refinement of scoring system: all authors; Scoring of images for validation and ICC analysis: Sarah E. Gullbrand, Alon Lai, Julie B. Engiles, Yejia Zhang, Steven Presciutti, Carol Muehleman, Matthew Pelletier, Carla Cunha, Marion Fusellier, Jordy Schol, Takashi Yurube, James Crowley, Yoshiki Takeoka. Original manuscript draft: Sarah E. Gullbrand; Critical revisions of manuscript and final approval of the current manuscript: all authors. Authors performing scoring for validation or who provided images for validation studies are listed alphabetically.

## Supporting information


**Supplemental Data 1** A copy of the survey sent to spine researchers.

## Data Availability

The data that support the findings of this study are available from the first author and corresponding author (Sarah E. Gullbrand, James C. Iatridis and Koichi Masuda, respectively) upon reasonable request.
