# Supplementary material for: Development of a standardized histopathology scoring system for intervertebral disc degeneration and regeneration in rabbit models‐An initiative of the ORSspine section
Source: JOR Spine. 2021 Jun 3;4(2):e1147. doi: 10.1002/jsp2.1147 (PMC8313151; doi:10.1002/jsp2.1147)
Supplement: Supplementary file 1 — Supplemental Data 1 A copy of the survey sent to spine researchers. [file JSP2-4-e1147-s001.pdf]

JOR Spine Histopathology Questionnaire - Rabbit Models

Please answer the following questions to help us develop a consensus histology scoring system for rabbit models of intervertebral disc degeneration/regeneration.

* Required

1. Which method of sectioning should be utilized for histology grading *

Mark only one oval.

- Paraffin
- Cryosectioning
- Methyl methacrylate (plastic)
- I don't know/don't have enough expertise

2. Which sectioning plane should be utilized for histology grading? *

Mark only one oval.

- Sagittal
- Coronal
- Axial/Transverse
- I don't know/don't have enough expertise

3. What thickness of the sections (micrometers) should be used for histology? *

4. Which stains should be used for histology grading (select all that apply)? *

Check all that apply.

- [ ] Hematoxylin and Eosin
- [ ] Safranin-O and Fast Green
- [ ] Alcian blue and Picrosirius Red
- [ ] Masson's Trichrome
- [ ] Van Gieson
- [ ] Toluidine blue
- [ ] I don't know/don't have enough expertise

Other: [ ] ______________________

5. Rate the importance of including the following category in a histology scoring system: Nucleus Pulposus (NP) Morphology *

Mark only one oval.

	1	2	3	4	5	
Least important	( )	( )	( )	( )	( )	Most Important

6. Rate the importance of including the following category in a histology scoring system: NP Cellularity *

Mark only one oval.

	1	2	3	4	5	
Least Important	( )	( )	( )	( )	( )	Most Important

7. Rate the importance of including the following category in a histology scoring system: Annulus Fibrosus (AF) Morphology *

Mark only one oval.

	1	2	3	4	5	
Least Important	◯	◯	◯	◯	◯	Most Important

8. Rate the importance of including the following category in a histology scoring system: AF Cellularity *

Mark only one oval.

	1	2	3	4	5	
Least Important	◯	◯	◯	◯	◯	Most Important

9. Rate the importance of including the following category in a histology scoring system: Border between the NP and AF *

Mark only one oval.

	1	2	3	4	5	
Least Important	◯	◯	◯	◯	◯	Most Important

10. Rate the importance of including the following category in a histology scoring system: Endplate *

Mark only one oval.

	1	2	3	4	5	
Least Important	◯	◯	◯	◯	◯	Most Important

11. Are there other categories which should be included in a histology scoring system?

____

12. Which features should be considered when scoring the category of "NP Morphology"? (select all that apply) *

Check all that apply.

- [ ] Intensity of proteoglycan staining
- [ ] Matrix condensation
- [ ] Shape of the NP
- [ ] I don't know/don't have enough expertise

Other: [ ] ______________________________

13. Which features should be considered when scoring the category of "NP/AF Cellularity"? (select all that apply) *

Check all that apply.

- [ ] Cell morphology
- [ ] Cell number
- [ ] Cell clustering
- [ ] I don't know/don't have enough expertise

Other: [ ] ______________________________

14. Which features should be considered when scoring the category of "AF Morphology"? (select all that apply) *

Check all that apply.

- [ ] Lamellar organization (U-shape, inward bulging)
- [ ] Tears/fissures/disruptions
- [ ] I don't know/don't have enough expertise

Other: [ ] ______________________________

15. Which features should be considered when scoring the category of "Endplate"? (select all that apply) *

Check all that apply.

- [ ] Cartilage endplate thickness
- [ ] Cartilage endplate cellularity
- [ ] Cartilage endplate disruptions
- [ ] Osteophyte formation
- [ ] Boney sclerosis
- [ ] I don't know/don't have enough expertise

Other: [ ] ______________________

16. How many points should be assigned to the range of degeneration within each category (i.e. 0-2, 0-3, etc) *

Mark only one oval.

( ) 3
( ) 4
( ) 5
( ) Other: ______________________

17. Which other study outcomes would you like to see included in future consensus methods papers for the rabbit model? Select all that apply. *

Check all that apply.

- [ ] Radiographs (Disc Height Index)
- [ ] MRI
- [ ] Biomechanical Testing
- [ ] microCT
- [ ] Biochemical assays (GAG, collagen content)
- [ ] Gene Expression
- [ ] molecular labelling of structures in the nervous system

Other: [ ] ______________________

18. Do you have any other suggestions to help us develop a standardized histology scoring system for rabbit models?

____
____
____
____
____

19. Are you willing to share images for validating the proposed rabbit degeneration scoring system? *

Mark only one oval.

- ◯ Yes
- ◯ No

20. Are you willing to be a grader for validating the proposed rabbit degeneration scoring system? *

Mark only one oval.

- ◯ Yes
- ◯ No

21. Name (only to be used in this survey & histopathology article) *

____

22. E-mail address (for survey follow up if you indicated you are willing to provide/grade images)

____

This content is neither created nor endorsed by Google.

Google Forms
